# Comparative evaluation of live attenuated and killed tachyzoites as vaccine candidates for toxoplasmosis

**DOI:** 10.1186/s13568-025-01889-3

**Published:** 2025-07-10

**Authors:** Eman E. El Shanawany, Eman H. Abdel-Rahman, Waleed A. Nemr, Soad E. Hassan, Noha M. F. Hassan, Hassan M. Desouky, Rabab Zalat, Amany Ebrahim Nofal, Raafat M. Shaapan, Salwa Sami Younis

**Affiliations:** 1 Parasitology and Animal Diseases Department, Veterinary Research Institute, National Research Centre, Dokki, Giza, Egypt; 2 Department of Radiation Microbiology, National Center for Radiation Research and Technology, Egyptian Atomic Energy Authority, Cairo, Egypt; 3 Animal Reproduction and Artificial Insemination Department, National Research Centre, Dokki- Giza, Egypt; 4 Department of Parasitology, Theodor Bilharz Research Institute, Giza, Egypt; 5 Zoology Department, Faculty of Science, Histology and Histochemistry, Menoufia University, Shibin El-Kom, Egypt; 6 Department of Zoonosis, Veterinary Research Division, National Research Center, Dokki, Giza, Egypt; 7 Medical Parasitology Department, Faculty of Medicine, Alexandria University, Alexandria, Egypt

**Keywords:** Gamma irradiation, Vaccine, Live attenuated tachhyzoites, *Toxoplasmagondii*, Mice, Acute toxoplasmosis

## Abstract

*Toxoplasma gondii* (*T.gondii*) an obligate intracellular protozoan, causes toxoplasmosis, leading to significant economic losses and posing serious public health challenges worldwide. Developing an effective vaccine for toxoplasmosis in humans remains difficult. In this study, we evaluated the protective immunity of tachyzoites local virulent strain exposed to 0.25 KGy as a live attenuated vaccine and 1.5 KGy as a killed vaccine. Swiss albino mice were immunized with three doses of each vaccine at 2-week intervals. Four weeks after the final immunization, mice were challenged with 2500 tachyzoites of RH HXGPRT (–) strain. Mice immunized with live attenuated vaccine showed a significant *p* < 0.05 increase in survival time (67.778 ± 26.4 days) compared to a control group, mice group immunized with killed tachyzoites, and the group injected with adjuvant. In the live attenuated vaccinated group, the mice percentage that still survived along six months of follow-up was 57.1% without any signs of acute toxoplasmosis. The infection reduction percentage was significantly higher (99.8%) in the live attenuated vaccine group, with the complete absence of tachyzoites in liver impression smears. Our results demonstrated that live attenuated vaccine triggered a strong immune response, marked by significantly elevated levels of IFN-γ, IL-12, and IL-17 cytokines, and increased percentages of CD4 ^+^ and CD8^+^ T-lymphocytes. High levels of *Toxoplasma*-specific IgG were maintained. Histopathological analysis showed a complete absence of the parasite in the liver and spleen of mice vaccinated with live attenuated vaccine, with hepatic cells appearing normal and moderate aggregations of inflammatory cells observed in the hepatic portal area. In conclusion, the comparison between the use of killed and live attenuated tachyzoites proved that the use of live gamma-irradiated attenuated tachyzoites is effective in eliciting cellular and humoral immune responses against acute toxoplasmosis in mice, presenting potential candidate prepared by conventional approach for the development of a vaccine against toxoplasmosis. The sheep isolate strain used in our study represents a virulent strain with characteristics similar to those that circulate in natural populations, which makes it an appropriate candidate for vaccine development in livestock and then for humans.

## Introduction

Foodborne zoonotic toxoplasmosis is the name for the apicomplexa intracellular protozoan parasite disease that is brought on by *T. gondii*. Numerous species, including humans, are thought to serve as their intermediate hosts, with the cat family being the final host (Moncada and Montoya 2012; Hassan et al. 2016; Milne et al.^2020^). *T.gondii* infects almost 50% of people on the planet (Weiss and Dubey 2009). In immunocompromised individuals, the disease is life-threatening, but in self-limiting and asymptomatic individuals, it presents with minor lymphadenopathy (Cañón-Franco et al. 2014; Assolini et al. 2017; Layton et al. 2023). Moreover, in veterinary medicine, it is a major factor contributing to newborn diarrhea in calves (Göhring et al. 2014; El Shanawany et al. 2024a, b), resulting in a substantial financial loss (Torgerson and Macpherson 2011).

Currently, no medication can fully cure the infected host and eliminate the chronic encysted form of toxoplasmosis. Furthermore, it can be associated with adverse effects and cannot prevent reinfection (Konstantinovic et al. 2019). The combination of pyrimethamine and sulfadiazine was used for treatment only acute stage of infection moreover, resistance against the drug usually occurs (Alday and Doggett 2017). Therefore, the discovery of a protective vaccine is the most potentially effective means for eradication of this parasitic infection (Foroutan et al.^2019^).

The only commercially available live attenuated S48 strain vaccine is called Toxovax, which has been approved for use in preventing congenital infection in sheep and goats. This vaccine, however, is not suitable for humans because it may evolve into a pathogenic strain. Furthermore, it is costly, has negative side effects, and has a short shelf life (Zhang et al. 2015; Innes et al. 2019). So, the development of an ideal vaccine is still a challenge, because of the complexities of the *T.gondii* life cycle, genome, and diversity of strain. Moreover, there remains a need for improved vaccines, especially those that target different strains or provide broader immunity.

Effective subunit protein vaccines demand the use of several strategies including delivery systems and adjuvants for protective, adequate, and efficient immune response. The uptake of protein antigens by antigen-presenting cells results in the activation of adaptive immune response. Unfortunately, most soluble proteins cannot be taken by antigen-presenting cells; this explains why weak immune responses are associated with the use of isolated proteins as vaccine candidates (Cardi et al. 1998).

Attenuation of the parasite was elicited by gamma irradiation, chemical treatment, gene knockout live-attenuated strain, and multiple passages resulting in the parasite being unable to complete its life cycle by reduction of virulence (Yang et al. 2019; Wang et al. 2020; Zhang et al. 2024). Simultaneously, the strain maintains its ability to provoke an immunological response in the host, enabling it to generate memory cells and thwart reinfection. Low-dose ionizing radiation is linked to mitotic mortality because it breaks double-stranded DNA and disrupts the daughter cell's chromosome. As a result, the parasite's ability to infect is diminished. However, the high doses of radiation result in the production of free radicals, which can cause direct destruction of the cell membranes (Szumiel 1994). Poor soluble protein aggregates were formed using ionizing radiation **(**Do Nascimento et al. 1996), which results in slow delivery systems as some Alum-type adjuvants act. The effective use of irradiation in vaccine development has been most recently demonstrated in the development of rapid vaccine candidates against the COVID-19 pandemic, caused by the virus SARSCoV-2. Gamma-inactivated SARS-CoV-2 vaccine candidate was able to elicit neutralizing antibodies and a strong T and B cell response (Sir Karakus et al.^2021^; Turan et al. 2022).

Irradiated protein extract of *T.gondii* tachyzoites induces good protection in mice with efficient humoral and cellular immune responses (da Costa et al. 2020). There are reports on experimental parasitic vaccines generated by ionizing irradiation, including *T.gondii* tachyzoites with promising results (Hiramoto et al. 2002; Zorgi et al. 2011; Finkensieper et al. 2023). Our previous study El Shanwany et al. (2024a) proved that the live attenuated tachyzoites exposed to the low dose of gamma irradiation 0.25 KGy and killed tachyzoites exposed to a high dose of gamma irradiation 1.5 KGy resulted in the election of humoral and cellular immune response without causing infection to the mice. So, the presented study aim to compare the protective effect of two different doses of gamma radiation (0.25, 1.5 KGy), evaluating the efficacy of both live attenuated and killed vaccines.

## Materials and methods

### Mice and ethical statement

Swiss albino mice aged 6–8 weeks and weighing 20–25 g were obtained from a colony at the National Research Centre (NRC), Egypt. Mice were housed under standard laboratory conditions (temperature 22 ± 2 °C, relative humidity 50–60%, and a 12-h light/dark cycle), with free access to standard rodent diet and water. Prior to the experiment, sera from all mice were tested by ELISA to confirm the absence of anti-*Toxoplasma gondii* IgG antibodies, ensuring no prior exposure to the parasite. All procedures followed the “Guide of Laboratory Animal Care” and “The Principles of the Ethics Committee of the Medical Research of NRC, Egypt” with approval number 13010125.

### Parasites

For the preparation of the immunizing agents tachyzoites were collected from the tissues of slaughtered sheep in Menoufiya Governorate, Egypt. Liver and heart tissues were cut into small cubes and placed in a digestive solution containing pepsin and HCl at 4 °C for a few hours until complete digestion as proved by light microscope (El-Nawawi et al. 2008). After digestion, the positive tissue samples will be intraperitoneally inoculated into Swiss albino mice with daily observation for signs of an ascites response that indicates acute toxoplasmosis in the mice (Ghazy et al. 2007), the maintenance of virulent isolated strain inoculation of mice was done according to Shaapan et al. (2023). The molecular detection and genotyping of the *T.gondii* isolate we have previously published, confirms the virulent nature of the isolated strain (Elfadaly et al. 2017).

For the infection challenge the RH HXGPRT (–) virulent strain of *T.gondii* strain (Donald and Roos, 1998) was maintained in Swiss Albino mice in the the laboratory of the Medical Parasitology Department, Faculty of Medicine, Alexandria University. Tachyzoites were isolated from the peritoneal fluid of previously infected mice on the 5th-day post-infection by intraperitoneal injection of euthanized mice with sterile saline followed by withdrawal of this saline. The number of tachyzoites in the collected peritoneal fluid was counted by hemocytometer and then, the dose was adjusted for infection at 2500 tachyzoites/100 μL saline /mouse (El-Kady et al. 2023).

### Irradiation of virulent *T.gondii* tachyzoites

The gamma irradiated vaccines were prepared by exposing aliquots of *T.gondii* tachyzoites to gamma rays. The irradiation process was carried out at roome temperature using the ionizing radiation emitted from Cobalt-60 isotope into a radiator facility (Gammacell-220 Excel 60Co irradiation Canadian Facility, established in NCRRT, EAEA, Cairo). The half amount of the irradiated aliquots were expose to 0.25 KGy for 19.48 min. (dose of radiation) for producing live attenuated tachyzoites vaccine, and the others were exposed to 1.5 KGy for 116.88 min. for producing killed tachyzoites vaccine. The non-irradiated aliquots were used in the subsequent challenge experiment. The dose rate at the time of this experiment was 0.77 KGy/h (El Shanawany et al. 2024a, b).

### Immunization and challenge in mice

In total, sixty mice were utilized in this experiment; they were split up into five groups, each with twelve mice. Group (Ia): Mice of control positive infected and non-vaccinated; group (Ib): Mice received Freund’s adjuvant (Sigma Immunochemicals, St Louis, MO, USA) complete in the first dose and incomplete in the second and third doses (Guobadia and Fabemi 1997) group (Ic): Mice vaccinated using live attenuated vaccine (2500 tachyzoites exposed to 0.25 KGy gamma radiation); and group (Id): Mice vaccinated with dead tachyzoites (2500 tachyzoites exposed to 1.5 KGy which was mixed with complete Freud’s adjuvant in the first dose and incomplete Freud’s adjuvant in the second and third doses) (El Temsahy et al. 2016); group (II): Mice of control negative (non-infected and non-vaccinated). The mice that were vaccinated with the killed tachyzoite vaccine received three subcutaneous injections spaced a two week apart. While those vaccinated with live attenuated tachyzoites were injected intraperitoneally at the same time interval.

**Fig. 1:**
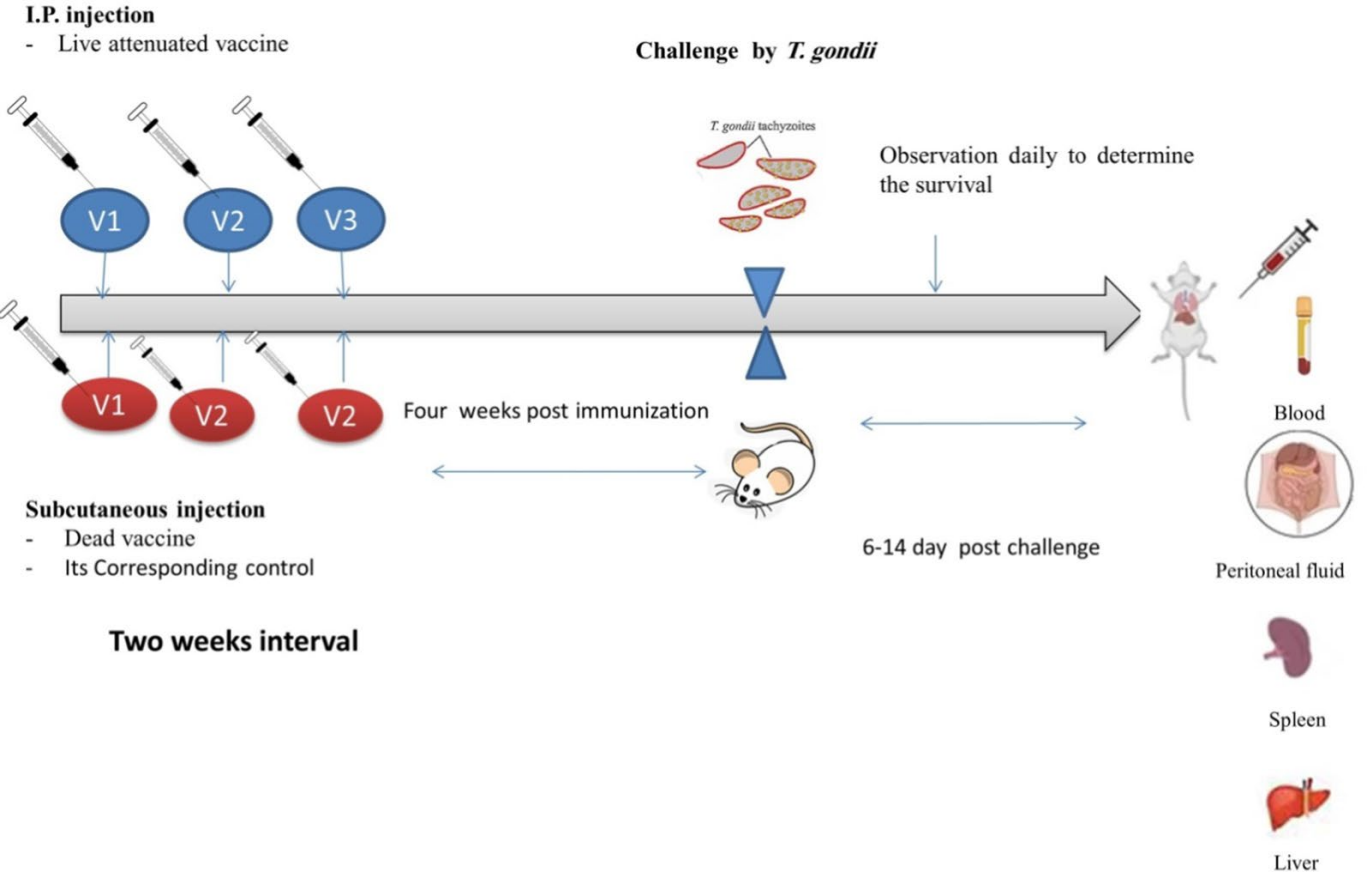
Infographic to show vaccination timings, sample collection, and processing

Four weeks after the last immunization dose, mice of the groups Ia, Ib, Ic, Id were challenged with the non-irradiated aliquots that contained 2500 tachyzoites of the virulent *T.gondii* strain. All groups were observed daily to determine the survival rate (Ezz Eldin et al. 2015**)** (Fig. 1). Half of the mice were euthanized by cervical dislocation at 6 days post-challenge, and the blood, liver, spleen, and peritoneal fluid from them were collected. Blood was collected from the study groups at 7, 16, 51, and 180 days post-vaccine. Serum samples were centrifuged at 4000 × g for 5 min, and then the sera were collected and stored at − 20 °C until further use.

## Assessment of vaccine efficacy

### Determination of tachyzoites number

To evaluate the parasite load in the mice after vaccination, the mean number of *T.gondii* tachyzoites in 1 ml of the peritoneal fluid was counted using a hemocytometer (HBGVR, Gießen, Germany) (El-Zawawy et al. 2015; Saad et al. 2023). The reduction percentage was calculated as the following equation:

$$ \begin{aligned}   {\text{Reductionrate}}\left( \%  \right)   = {\text{Tachyzoite count of control infected group}}/{\text{ml}} \\      \quad  - {\text{Tachyzoite count of control infected group}}/{\text{ml}}) \\    \quad /{\text{tachyzoite countofcontrol infected}}/{\text{ml}}) \times 100. \\  \end{aligned}  $$

In the liver impression smears the mean number of *T*. *gondii* tachyzoites was counted using a light microscope (El-Kady et al. 2023).

### Estimation of the survival rate and survival time

The survival time and clinical signs of toxoplasmosis in all tested mice groups were observed daily. The rate and time of survival for each group were recorded according to Eissa et al. (2012). Survival rate was calculated as the number of survived mice at the sacrifice time divided by the number of mice at the beginning of the experiment × 100.

### Rectal thermometry

The body temperature of vaccinated mice was measured weekly using Generic Digital thermometer Model H18593, with particular attention to the days following the first, second, and third vaccine doses. Temperature measurements were also taken after the challenge experiment and then weekly until eight weeks post-vaccination. Rectal thermometry, the standard method for measuring body temperature in rodents, provided colonic temperatures (Donhoffer 1980).

### Antigen preparation

*T.gondii* tachyzoites antigen was prepared as described by Yap (1983), Hassan et al., (2012). Briefly, peritoneal fluid was collected from the peritoneal cavity of infected mice at 6th. The collected fluid was then centrifuged and sediment was then resuspended in 0.9% sodium chloride then incubated in a shaking water bath to destroy red blood cells. The final suspension was filtered using a disposable syringe (25 ml, needle size 27) to release the tachyzoites from peritoneum cells. The tachyzoites were then washed in phosphate-buffered saline (PBS, pH 7.2) three times by centrifugation, the antigen would be sonicated several times in an ice bath for 20 s each time at 100 mAmp and the soluble antigen was collected after centrifugation at 12,000 rpm for 30 min. in a cooling centrifuge. The protein content was determined according to Lowry et al. (1951). The antigen was aliquoted and stored at − 20 °C until it was used.

### Measurement of specific *Toxoplasma* IgG antibody responses

The optimal concentrations of antigen, serum, and conjugate dilutions were determined using a checkerboard titration. The ELISA was conducted according to Connick et al. (2023) and Hegazi et al. (2023) with slight modifications which was change in antigen concentration used and time of incubation of sera and conjugates. Briefly, The ELISA plate was coated with 20 μg/ml of antigen and incubated overnight at 4 °C. The plate was then washed at least three times with Tween 20 PBS, pH 7.2. Blocking buffer was then added and incubated for 1 h at room temperature. The plate then was washed thrice. The tested sera were added at a dilution of 1:100 and incubated for 1.5 h at 37 °C, followed by another wash. The secondary anti-mouse IgG horseradish peroxidase (Prod. No. A4416, Sigma, USA) was then added at a dilution of 1:1000, and the plate was incubated at 37 °C for one hour. Color development was achieved by adding orthophenylenediamine (Prod. No. P23938, Sigma, USA) with 0.05% H2O2 (100 μl per well). The developed color was measured at 405 nm using an automatic micro ELISA reader (ELx 800, BIOTEK Instruments, Inc., Germany). The cut-off value was calculated and subtracted from all measured values (El Shanawany et al. 2023; Hassan et al. 2024).

### Measurement of cytokine response

IFN-γ, IL-17, and IL-12 concentrations were measured in tested mice sera according to the instruction of manufacture using commercially available sandwich ELISA kits purchased from Inova biovision, Beijing, China. The corresponding catalogue numbers were In-Mo1233, In-Mo1250, and In-Mo1244 respectively.

### Histopathological examinations

Liver and spleen tissue specimens were quickly removed from each mouse and fixed in 10% neutral buffered formalin. The tissues were then embedded in paraffin, cut into 4–5 µm sections, and placed on slides. For light microscopic examination, the sections were stained with hematoxylin and eosin (HE) stain (Bancroft and Gamble 2008).

### Immunohistochemical (IHC) analysis

The immunohistochemical avidin–biotin conjugate (ABC) method was performed after antigen activation of CD4^+^ and CD8^+^ (Cluster of differentiation 4 & 8, T-cell receptors) and blocking non-specific binding in deparaffinized mice liver Sects. (3–4 µm) to detect specific CD4^+^ T helper lymphocytes, and cytotoxic CD8 ^+^ T cells that co-localized with hepatic stellate cells (HSCs) by using Anti CD4^+^ Antibody (D7D2Z, Cat No: 25229, Rabbit mAb-Cell Signaling Technology (CST), IHC antibody Dilution: 1:80, company headquartered in Danvers, Massachusetts, USA) (Adam and Mor 2022); and Anti-mouse CD8^+^ antibody (Cat No: sc-70802, IHC antibody Dilution: 1:50, Santa Cruz, California, CA, USA) (Koda et al. 2021), which showed membrane and cytoplasmic staining of cells. One drop of DAB (3,3' Diaminobenzidine) per milliliter of substrate buffer was used (100 μL of DAB working solution for each section) and produced a brown precipitate at the site of the CD4^+^ and CD8^+  ^antigens. The slides were washed with deionized water when the desired staining developed a brown color and then counterstained by using Mayer’s hematoxylin. The slides were examined and scanned by utilizing an Olympus BX 41 microscope. Reacted brown-stained cells were counted in 5 fields at × 40 magnification of each liver section of mice (n = 5) by using Image J, averaged, and expressed as mean ± standard division (López et al. 2014; Naiket al. 2015).

### Data analysis

The obtained results were expressed as mean ± standard error. The level of significance was set at a value of *p < 0.05**,** **p < 0.001 ***p < 0.0001 using ordinary one-way ANOVA, Tukey's multiple comparisons test was used. Survival after challenge with the virulent *Toxoplasma* strain was expressed using the Kaplan–Meier curve. The Statistical analysis was performed, and graphs were plotted using MedCalc and GraphPad Prism version 6.0 (GraphPad Software, La Jolla, CA, USA). The normality of data distribution was assessed using the Shapiro–Wilk test. The results demonstrated that all groups (Ia, Ib, Ic, Id) followed a normal distribution (*p* > 0.05 for all groups). Therefore, parametric statistical analyses, including one-way ANOVA, were appropriately applied.

## Results

### Survival time

**Fig. 2:**
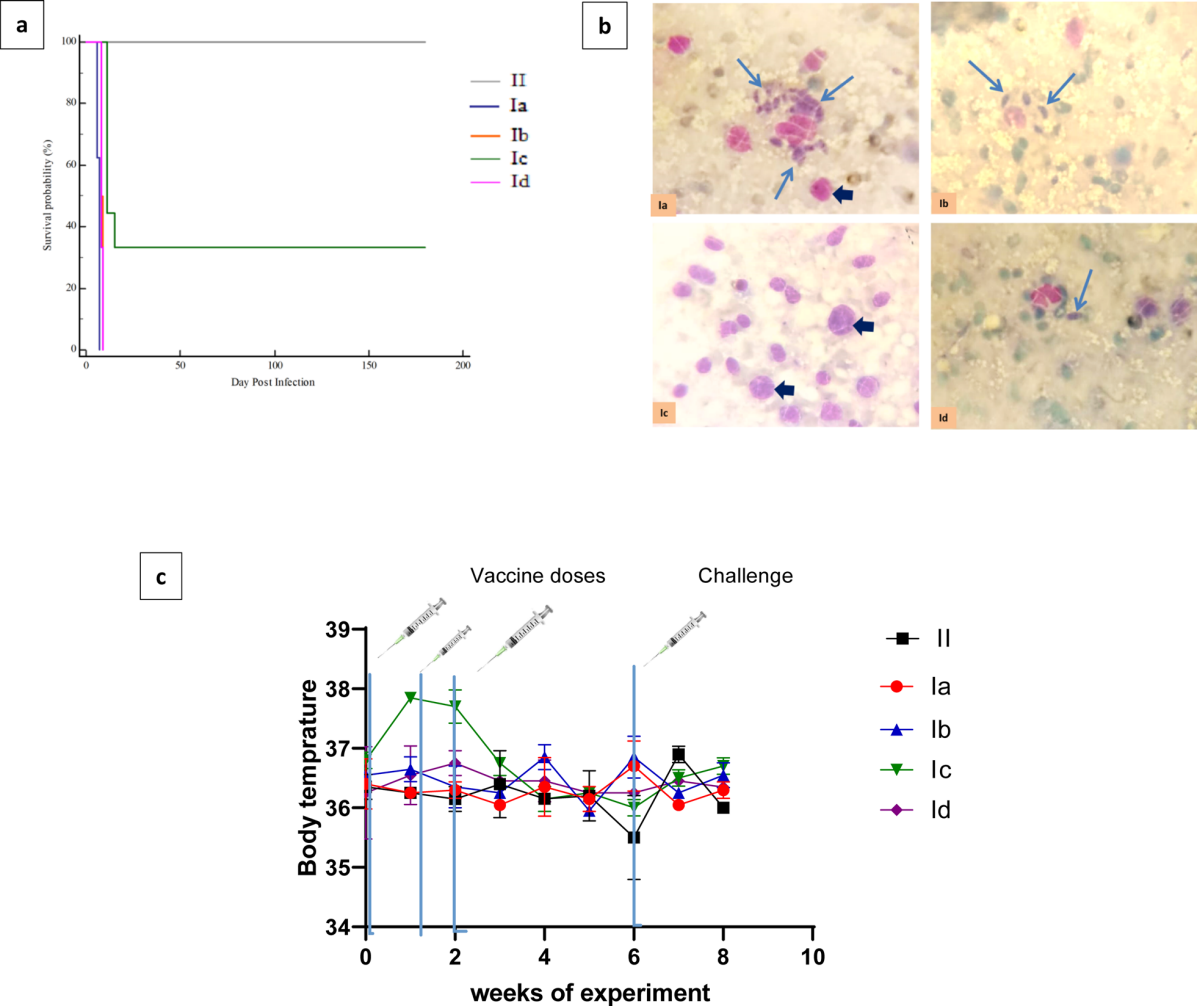
**a** Kaplan–Meier overall survival curve for the studied groups. **b **Liver impression smears from tested groups.** c** Rectal body temperature of the mice following during the experiment period. II: Control negative (non-infected non-vaccinated group). (Ia) control positive (Infected non-vaccinated group). Ib: Control group that received adjuvant complete and incomplete. Ic: Mice vaccinated with prepared live attenuated vaccine. Id: Mice vaccinated with the dead-prepared vaccine.. Data are presented as means ± standard error

According to daily monitoring of mouse survival, the mice in the infected control group (Ia), were dead within 7 days, with a mean survival time of 6.625 ± 0.183 days. In contrast, the mice vaccinated with a live, attenuated, prepared vaccine displayed a mean survival time of 67.778 ± 26.4 days. While the mice immunized with the dead-prepared vaccine exhibited 8.333 ± 0.211 survival time with a 25% survival rate. The group that received adjuvant displayed a mean survival time of 8.500 ± 0.189 (Fig. 2a, Table 1). Startlingly, in group Ic the mice percentage that still survived along six months of follow-up was 57.1% without any signs of acute toxoplasmosis. The results of the examination of the remaining surviving mice at 180 dpi to look for cysts demonstrated that there are none in the brain.

**Table 1:** The time of survival in mice of different studied groups

Groups	Mean ± SE	*P* value	95% CI for the mean
II (normal non infected non vaccinated)	180.000 ± 0.000	*p < *0.0001*	180.000 to 180.000
Ia (control infected non-vaccinated)	6.625 ± 0.183		6.266 to 6.984
Ib(control mice received adjuvant complete and incomplete)	8.500 ± 0.189		8.130 to 8.870
Ic (mice vaccinated with live attenuated vaccine)	67.778 ± 26.4		15.928 to 119.628
Id (mice vaccinated with dead vaccine)	8.333 ± 0.211		7.920 to 8.747

^*^ Significant *p < 0.05**,** **p < 0.001 ***p < 0.0001

### Parasite burdens

There was a statistically significant decrease in the mean number of tachyzoites in the peritoneal fluid of vaccinated groups (Ic and Id), as compared with a control-infected non-vaccinated group (Ia) and a control group that received adjuvant (Ib). The lowest mean number was achieved in group Ic which was vaccinated with prepared live attenuated vaccine (0.33 ± 0.21) and exhibited the most effective reduction of *T.gondii* tachyzoites (Table 2).

**Table 2:** Percent of reduction and mean count of *T. gondii* tachyzoites in mice peritoneal fluid of all studied groups

Groups (n = 6)	Meanx10^3^ ± SE	%reduction	*P* value	Pairwise comparison
Ia (control infected non-vaccinated)	457.5 ± 61.51		*P < 0.0001	P1** = 0.0030,P2*:0.0001, P3*** = 0.0001,P4*:0.0001, P5*: < 0.0001, P6* = 0.05
Ib (control mice received adjuvant complete and incomplete)	265.7 ± 24.71	45		
Ic (mice vaccinated with live attenuated vaccine)	0.3333 ± 0.21	99.8		
Id (mice vaccinated with dead vaccine)	6.667 ± 3.5	97		

*Significant* *p < 0.05**,** **p < 0.001 ***p < 0.0001*; P1*: I vs. Ib, *P2:* I vs. Ic, *P3*: I vs. Id, *P4*: Ib vs. Ic, *P5*: Ib vs. Id, *P6*: Ic vs. Id

Moreover, the tachyzoite number was counted in liver impression smears. The results showed a significant decrease in the number of tachyzoites in the Id and Ib groups, which are 3.167 ± 1.276 and 6.333 ± 1.476 respectively, in comparison with the control infected non-vaccinated (20.50 ± 4.193) (*p* < 0.0001). However, group Ic showed a complete absence of tachyzoites (Table 3, Fig. 2b).

**Table 3:** *Toxoplasma gondii* tachyzoites mean count in liver impression smear of different studied groups of mice

Groups (n = 6)	Meanx10^3^ ± SE	*P* value	Pairwise comparison
Ia (control infected non-vaccinated)	20.50 ± 4.193	*P < 0.0001	P1** = 0.0028, P2***: < 0.0001, P3** = 0.0003
Ib (control mice received adjuvant complete and incomplete)	6.333 ± 1.476		
Ic (mice vaccinated with live attenuated vaccine)	0.000 ± 0.000		
Id (mice vaccinated with dead vaccine)	3.167 ± 1.276		

*Significant* *p < 0.05**,** **p < 0.001 ***p < 0.0001*; P1*: I vs. Ib, *P2:* I vs. Ic, *P3*: I vs. Id

### Effect of the vaccine on the body temperature of mice

The body temperature of the mice was monitored to detect potential inflammation or unusual side effects (Fig. 2c). Following the first comparison with Ia group there was a slight but not significant increase in the core body temperature of the vaccinated mice groups (Ib,Ic,Id). Group Ib recorded a temperature of 36.45 °C ± 0.35, which was a slight increase but not significant in comparison with a control noninfected non vaccinated group. However, group Ic exhibited a slightly higher temperature (37.7 °C ± 0.281) compare to Ib, indicating a significant rise. After the second and third doses of the vaccine, group Ic showed a slight but non-significant elevation with temperatures recorded at 36.5 °C ± 0.286 and 36.75 °C ± 0.21, respectively in comparison with the control noninfected non vaccinated group (36.15 ± 0.212, and 36.4 ± 0.56). Group Id also showed non significant increase in body temperature following the vaccination. These findings indicate that no significant adverse effects on body temperature were detected post-vaccination.

### Effect of vaccine on mice humoral immune response

**Fig. 3:**
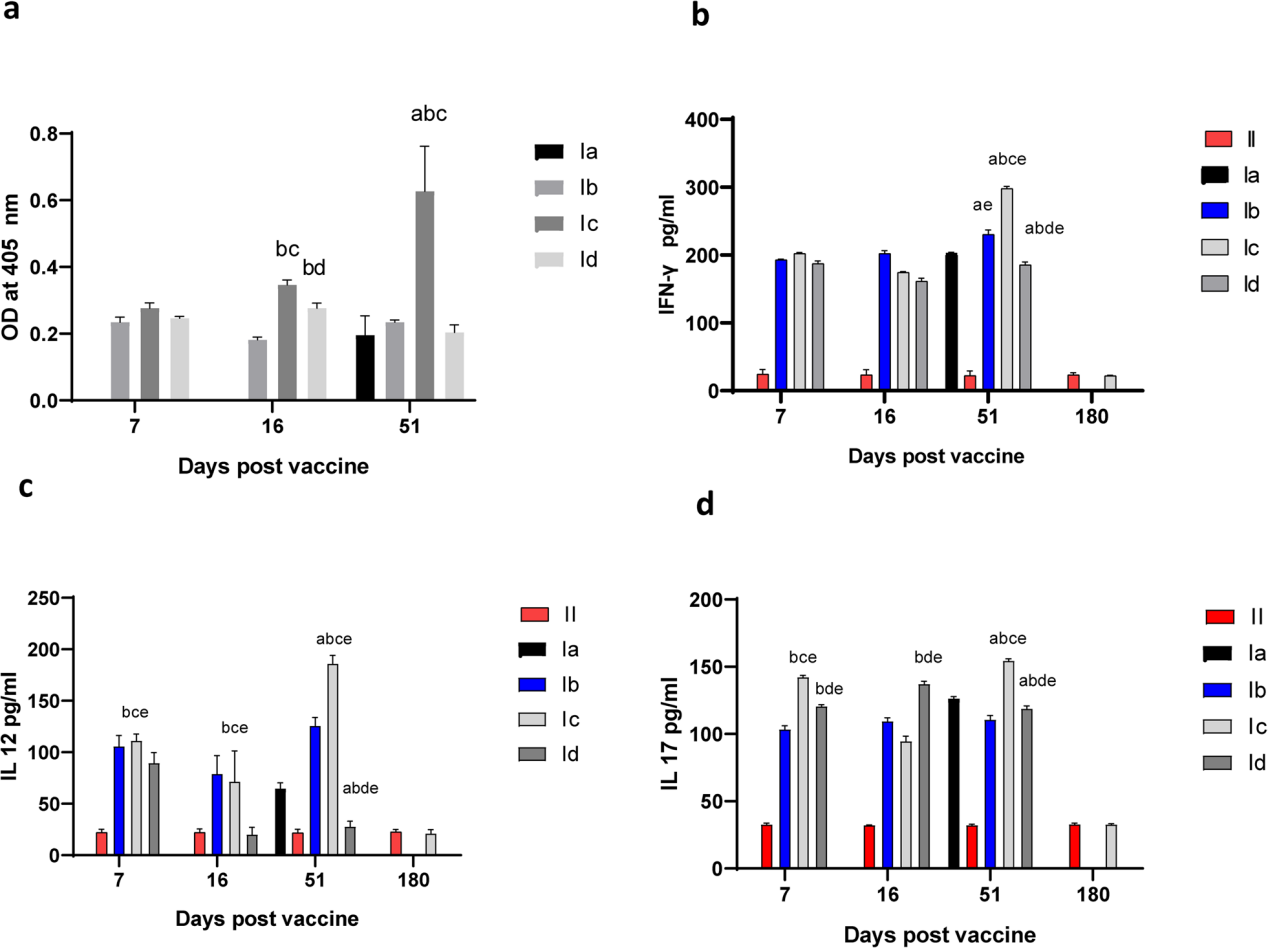
The serum levels of **a** anti-*Toxoplasma* IgG in the studied groups **b** IFN-γ, **c** IL-12, and **d** IL-17 in all studied groups. II): Normal non-infected non-vaccinated group Ia): Control infected none vaccinated. Ib): Control mice received adjuvant complete and incomplete. Ic): Mice vaccinated with live attenuated vaccine. Id): Mice vaccinated with a dead vaccine. The cytokines were quantified using ELISA. Each bar represents the mean OD ± S.E. The superscripted letters ^a, b, c, d, e^ indicate significance against infected control (Ia), adjuvant-treated (Ib), and vaccinated with dead vaccine (Id), vaccinated with dead vaccine (Ic), control normal non vaccinated non infected respectively

Significantly high levels of anti-*T.gondii* IgG was recorded in mice vaccinated with a live attenuated vaccine (Ic) at 7, and 16-days post-vaccination. The IgG level in this group was increased remarkably (*p* < 0.05) at 51 days post-vaccine (6 days post-challenge) (Fig. 3a) in comparison with control-infected non-vaccinated mice, indicating that a robust humoral response was conducted. However, the vaccination with the dead prepared vaccine (Id) and the group that received adjuvant (Ib) did not show a significant difference in comparison with the control infected non-vaccinated one at 51 days post-vaccination.

### Effect of the vaccine on mice cellular immune response

The IFN-γ, IL-12, and IL-17 levels in immunized groups Ic and Id showed a significant increase (*p* < 0.05) at 16, 51 days post-vaccine with a peak level at 51 days post-vaccine This is in comparison with control infected non-vaccinated group (Ia). Meanwhile, the levels of these cytokines were decreased to reach the level of cytokines in normal mice at 180 days post-vaccination. Although the cytokine response profiles in groups Ia and Ib were generally similar, the Ib group tended to produce slightly lower or comparable peak levels of IFN-γ and IL-17, suggesting a comparable but less pronounced inflammatory response. The data suggest that the vaccine effectively induces a robust cytokine response in vaccinated groups (Ic and Id) (Fig. 3).

### Histopathological results

**Fig. 4:**
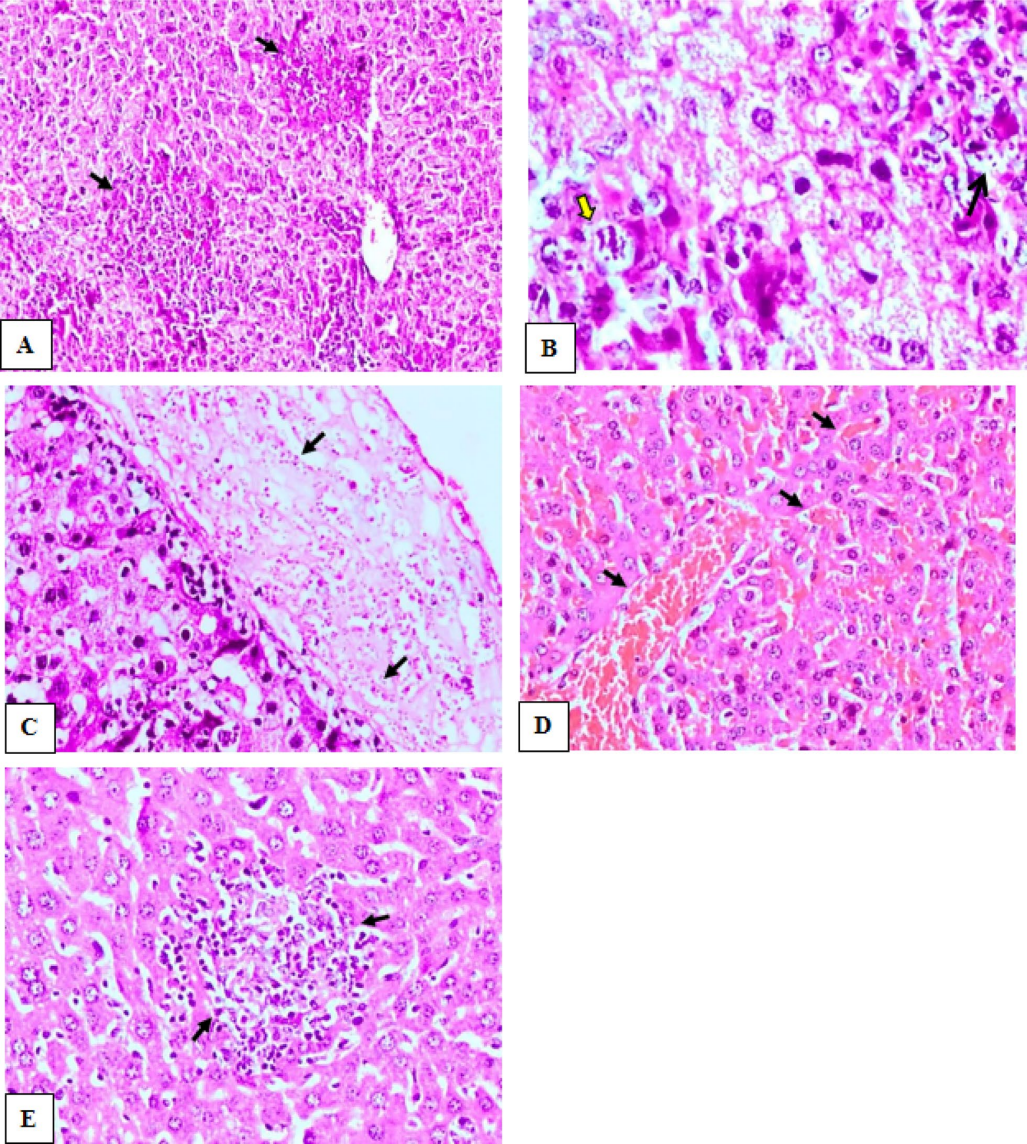
The results of H &E liver histopathological examination of control infected non-vaccinated group and live attenuated vaccinated mice. **A** Liver of group (Ia), showing multifocal areas of extensive hepatic cell necrosis (arrows) associated dilatation and congestion of hepatic blood vessels, vacuolar degeneration of adjacent hepatocytes (H&E, X200). **B** higher magnification of fig (A) showing the presence of tachyzoites pseudocyst (yellow arrow), free tachyzoites (black arrow), hepatocyte vacuolar degeneration (H&E, X400). **C** Liver of group (Ia), showing fibrinous perihepatitis(arrows) associated with high infiltration of free tachyzoites (H&E, X200). **D** Liver of group (Ia), showing marked dilatation and congestion of hepatic blood vessels and sinusoids (arrows) (H&E, X200). **E** Liver of group (Ic) showing, focal aggregations of inflammatory cells (arrows) associated with hepatic cell necrosis, and activation of kupffer cells (H&E, X200)

The histopathological results of the liver of the control-infected non-vaccinated group (Ia) showed the hepatic parenchyma showed multifocal areas of extensive hepatic cell necrosis associated with infiltration of free clusters of tachyzoites and pseudocysts. The adjacent hepatocytes showed vacuolar degeneration, in addition to scattered individual hepatic cell necrosis throughout the parenchyma. (Fig. 4A, B). Diffuse fibrinous perihepatitis associated with high infiltration of tachyzoites was observed (Fig. 4C). There was necrosis of periportal hepatocytes associated with massive aggregation of *T.gondii* tachyzoites. Dilatation of the bile duct associated with focal biliary epithelium hyperplasia was found. There were prominent dilatation and congestion of hepatic blood vessels, in addition to sinusoidal congestion was also seen (Fig. 4D).

The liver of mice vaccinated with live attenuated tachyzoites (Ic) revealed that hepatic cells appeared within the normal limit whereas they appeared polyhedral in shape, regularly arranged with granular eosinophilic cytoplasm and central vesicular nuclei. However, multiple foci of inflammatory cellular infiltration including mainly lymphocytes, macrophages, and a few neutrophils, associated with single hepatic cell necrosis throughout the hepatic parenchyma were noticed (Fig. 4E). Additionally, marked activation of Kupffer cells was seen. The hepatic sinusoids were relatively dilated and infiltrated with lymphocytic cells. The hepatic portal area showed mild to moderate aggregations of inflammatory cells. The hepatic blood vessels were dilated and congested.

**Fig. 5:**
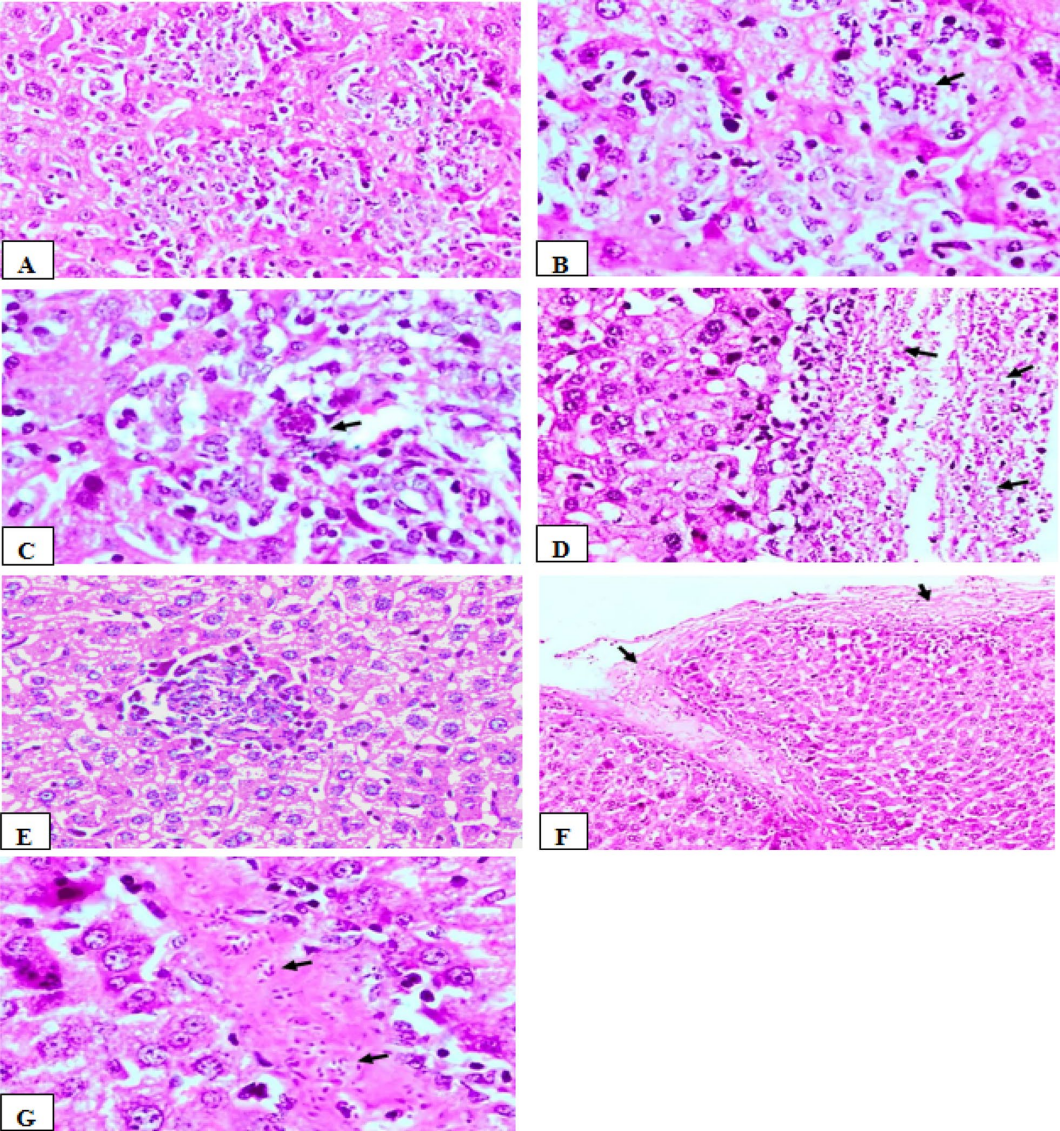
The results of H &E liver histopathological examination of vaccinated mice with dead tachyzoites and control adjuvant groups. **A** Liver of group (Id), showing diffuse and multifocal aggregations of inflammatory cells associated with hepatic cell necrosis and the presence of clusters of tachyzoites (arrow). (H&E, X200). **B** higher magnification of fig. (A), showing the presence of free clusters of *T. gondii* tachyzoites (arrow) (H&E, X400). **C** Liver of group (Id), showing, focal area of inflammatory cell infiltration associated with the presence of single tachyzoites pseudocyst (arrow) and hepatic cell necrosis. (H&E, X400). **D** Liver of group (Id), showing fibrinous perihepatitis(arrows) associated with thickening of the capsule and presence of massive numbers of free tachyzoites (H&E, X200**)**. **E** Liver of group (Ib) showing focal aggregation of inflammatory cells associated with hepatic cell necrosis, and degenerative changes of neighboring hepatocytes (H&E, X200). **F** liver of group (Ib) showing fibrinous perihepatitis(arrows) associated with the presence of tachyzoites, and subcapsular hepatic cell necrosis (H&E, X100). **G** Liver of group (Ib) showing the presence of free clusters of *T.gondii* tachyzoites (arrows) within fibrinous eosinophilic, proteinaceous material (H&E, × 400)

In the liver of mice vaccinated with dead tachyzoites (Id) group, liver sections showed diffuse and multifocal infiltrations of mononuclear inflammatory cells associated with widespread hepatic cell necrosis. Clusters of free tachyzoites and pseudocysts were evident (Fig. 5A–C). Focal mild interstitial hemorrhages and sinusoidal leukocytosis were also observed. The portal areas showed moderate lymphocytic infiltration, and focal fibrinous perihepatitis with numerous tachyzoites was detected (Fig. 5D). Hepatic blood vessels were congested.

However, in the group of mice injected with adjuvant (Ib), the liver showed multifocal aggregations of inflammatory cells associated with hepatic cell necrosis.Additionaly, clusters of tachyzoites were also seen (Fig. 5E). The hepatocytes revealed degenerative changes including granular and vacuolar type. Diffuse fibrinous perihepatitis associated with the presence of numerous tachyzoites was detected (Fig. 5F). Moreover, focal deposition of fibrinous eosinophilic, proteinaceous material (exudate) associated with the presence of numerous free *T.gondii* tachyzoites was noticed (Fig. 5G). The hepatic blood vessels appeared dilated and congested.

**Fig. 6:**
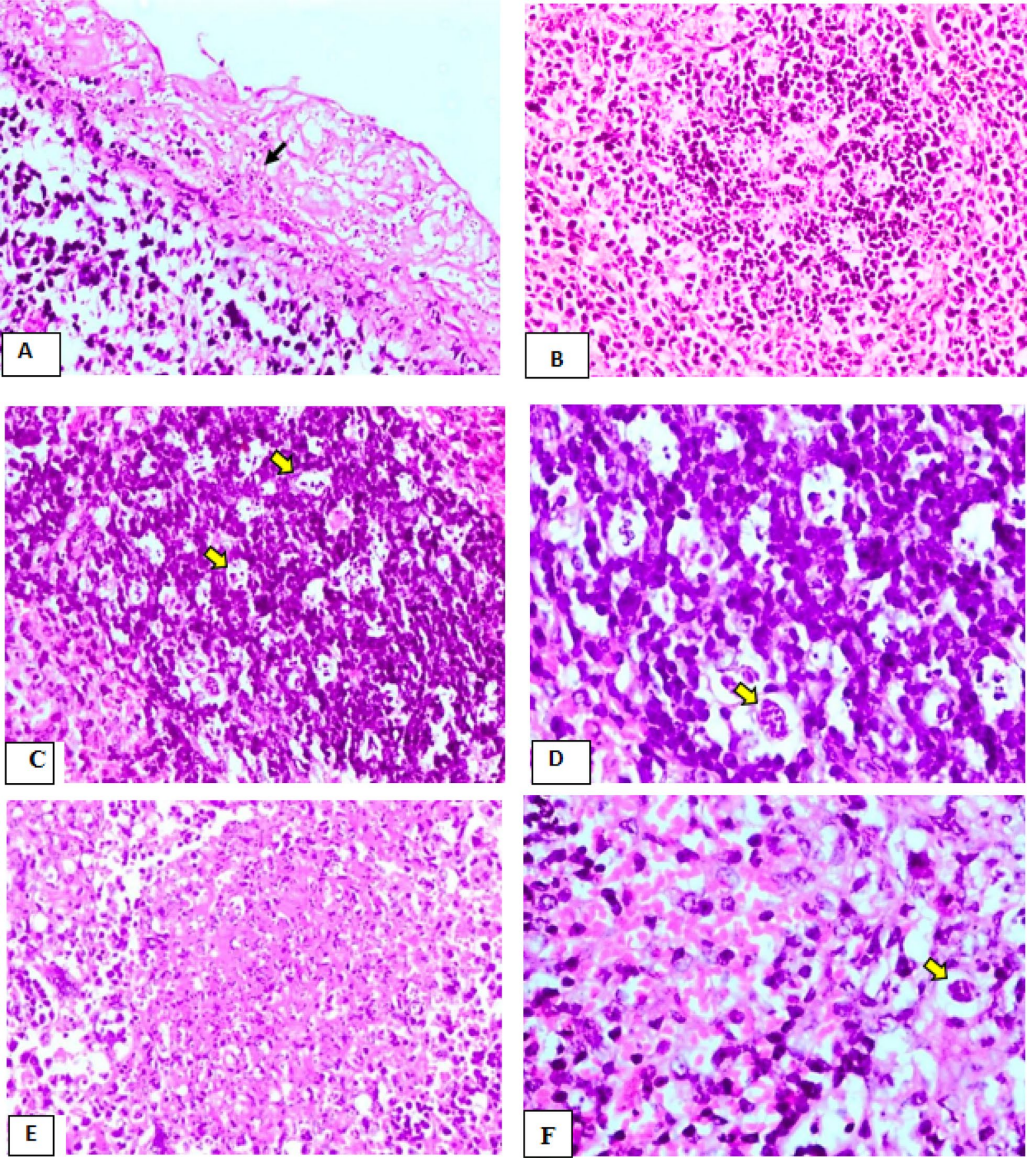
The results of H &E spleen histopathological examination of the control infected non-vaccinated group (Ia).** A** showing fibrinous perisplenitis with marked thickening of the capsule and the presence of massive numbers of tachyzoites(arrow) (H&E, X200). **B** showing severe lymphocytic cell depletion and necrosis of lymphoid follicles of white pulp (H&E, X200). **C** showing multiple scattered lymphocyte apoptosis & tingible body macrophages (yellow arrows) and tachyzoites pseudocyst in the germinal center of the lymphoid follicle (H&E, X200). **D** Higher magnification of fig. (C), showing multiple scattered apoptotic lymphocytes surrounded by clear space and tachyzoite pseudocyst (yellow arrow) in the germinal center of lymphoid follicle (H&E, X400). **E** showing the focal area of extensive necrosis in the red pulp (H&E, X100). **F** Showing the presence of tachyzoite pseudocyst in the sinuses of the red pulp(yellow arrow) (H&E,X200)

The spleen of control infected non-vaccinated group (Ia) exhibited multifocal fibrinous perisplenitis with prominent thickening of the capsule and the presence of massive numbers of tachyzoites (Fig. 6A). The white pulp showed severe lymphocytic cell depletion and necrosis of the lymphoid follicles (Fig. 6B). Furthermore, multiple scattered clusters of apoptotic lymphocytes and tingible body macrophages within the germinal centers were also observed. Additionally, multiple tachyzoites pseudocysts were seen (Fig. 6C, D). The red pulp showed multifocal areas of necrosis associated with the presence of clusters of tachyzoites pseudocysts (Fig. 6E, F).

**Fig. 7:**
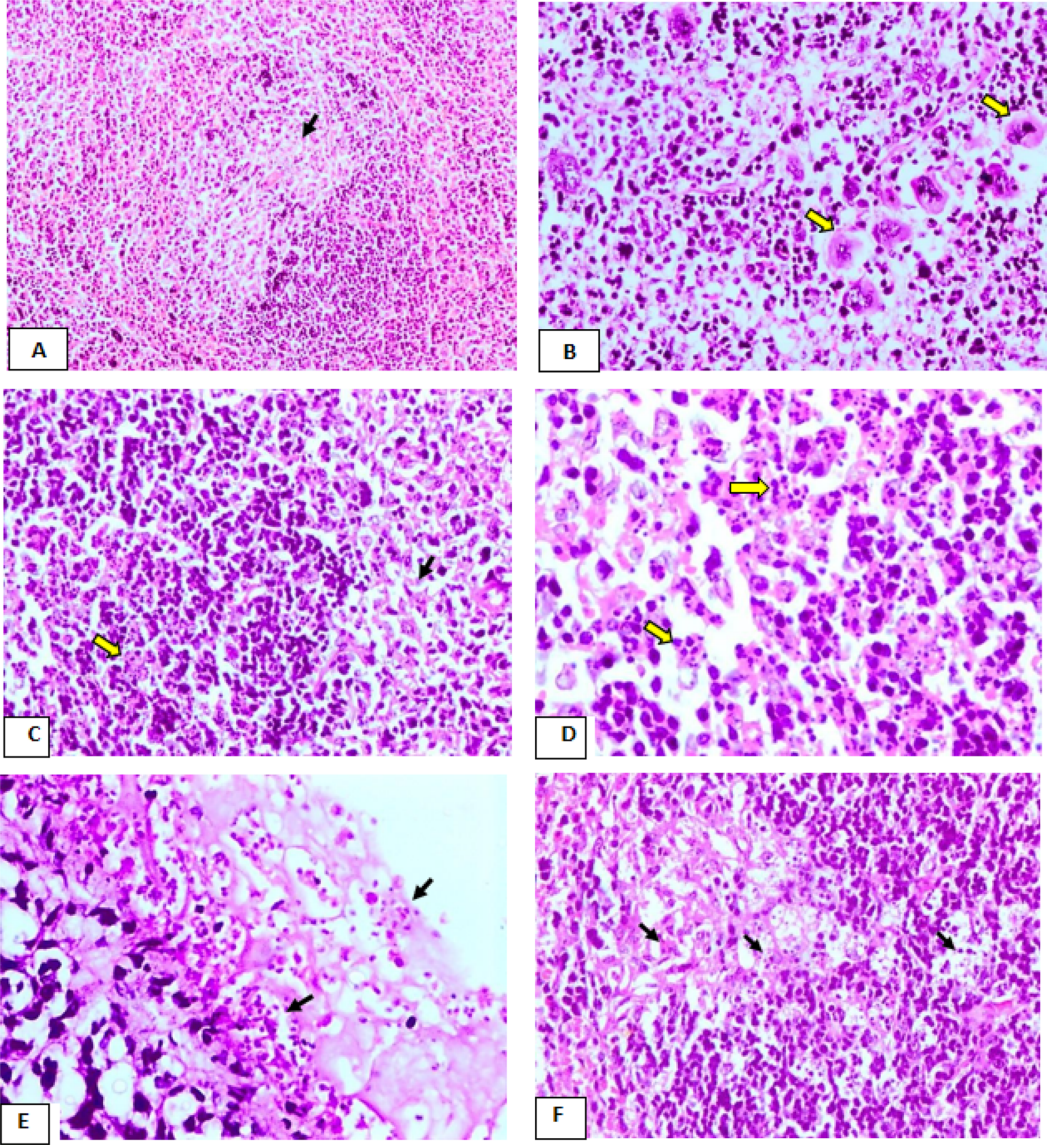
The results of H &E spleen histopathological examination of groups (Ic, Id, Ib). **A** Spleen of the group (Ic), showing moderate lymphoid depletion (arrow) and partial replacement of germinal center by faint homogenous eosinophilic fibrillated material and follicular dendritic cells (H&E, X100). **B** Spleen of the group (Ic), showing clusters of megakaryocytes(yellow arrows) in the sinuses of red pulp (H&E, X200). **C** Spleen of the group (Id), showing severe lymphocytic cell necrosis represented in the forms pyknosis, karyorrhexis, and karyolysis in the germinal center of the lymphoid follicle (yellow arrow), in addition to deposition of fibrillated eosinophilic substance intermingled with follicular dendritic cells and macrophages (black arrow) (H&E, X100). **D** Higher magnification of fig. **C** showing lymphocytic cell necrosis represented in the forms pyknosis & karyorrhexis (yellow arrows) (H&E, X400). **E** Spleen of the group (Ib), showing fibrinous perisplenitis associated with the presence of massive numbers of tachyzoites (arrows) (H&E, X400). **F** Spleen of the group (Ib), showing, severe lymphocytes necrosis associated with deposition of homogenous eosinophilic fibrillated material (arrows) intermingling with follicular dendritic cells and macrophages (H&E, X200)

The spleen of mice immunized with live attenuated tachyzoites group (Ic) showed moderate lymphocytic cell depletion associated with the deposition of faint homogenous eosinophilic fibrillated material in the germinal center of some lymphoid follicles of white pulp (Fig. 7A). However, follicular hyperplasia were recorded in few cases. Numerous megakaryocytes in sinuses of red pulp were also seen (Fig. 7B). However, the spleen of mice vaccinated by dead tachyzoites (Id) revealed moderate to severe lymphocytic cell necrosis represented in the forms of pyknosis, karyorrhexis, and karyolysis in most lymphoid follicles of white pulp was seen. Moreover, the germinal center of some follicles showed mild deposition of homogenous eosinophilic fibrillated substance intermingling with follicular dendritic cells and macrophages (Fig. 7C, D). A few numbers of free tachyzoites in both red and white pulp were noticed. The spleen of the Ib group of mice that received adjuvant(Ib) revealed multifocal fibrinous perisplenitis with marked thickening of the capsule and the presence of massive numbers of tachyzoites (Fig. 7E). There was severe lymphocytic cell necrosis represented in the forms of pyknosis and karyorrhexis associated with the presence of homogenous eosinophilic fibrillated substance intermingling with follicular dendritic cells and macrophages in the germinal center in the most lymphoid follicle of white pulp (Fig. 7F). Moreover, there was a remarkable increase in the number of megakaryocytes in the sinuses of the red pulp.

The histopathological findings of liver and spleen of both dead vaccine and group of mice injected with adjuvant were characterized by inflammatory cellular infiltration, hepatocellular necrosis, lymphocytic necrosis of lymphoid follicles of white pulp of spleen as well as presence of numerous tachyzoites. Such changes were severe in comparison to that of attenuated live vaccine group whereas the histopathological picture was significantly reduced and appeared mild to moderate, associated with absence of tachyzoites within the hepatic and splenic tissue.

### Immunohistochemical observations of CD4^+^ and CD8^+^ cellular immune response

**Fig. 8:**
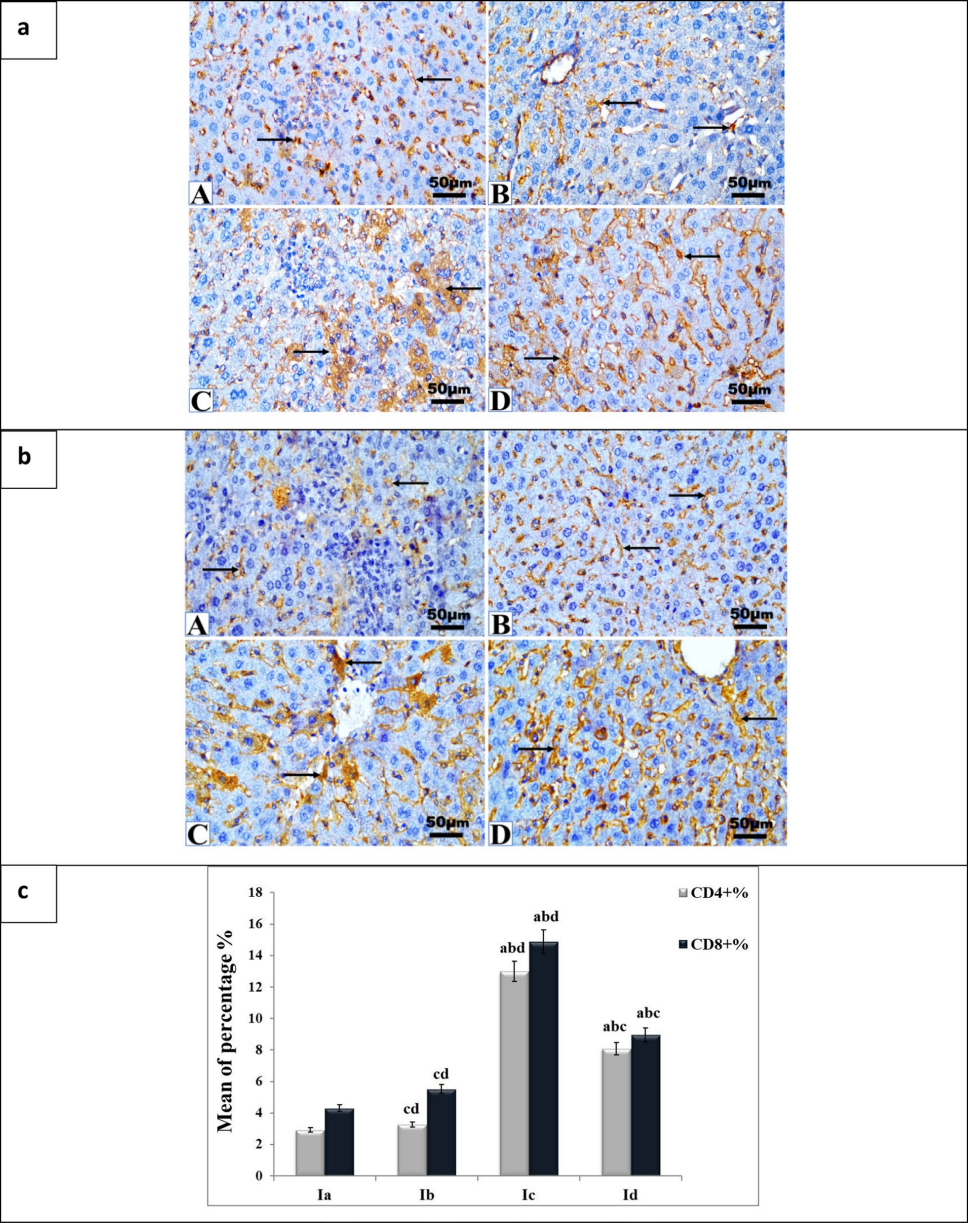
**a** CD4^+^ T-cells cellular immune responses in mice liver after different treatments., **b** CD8^+^ T- cellular immune responses in mice liver after different treatments. A–D are representative photographs obtained from IHC. Liver sections of the positive infected control group Ia (A), adjuvant-treated group Ib (B) vaccinated with killed tachyzoites Id (C), and (D) vaccinated with live attenuated tachyzoites Ic, showing positive brown immune expression of CD4^+^ T cells (arrows). IHC, × 40 magnification, bar 50 µm. **c **Data of the immune expression of CD4^+^ and CD8^+^ cells (%) in all the liver sections of the current groups are expressed as mean ± SD, n = 5, Ia control infected non-vaccinated, Ib adjuvant-treated, Ic mice group vaccinated with live attenuated tachyzoites, and Id vaccinated with dead tachyzoites vaccine. The superscripted letters ^a, b, c^ indicate *P* < 0.01 significance against infected control (Ia), adjuvant-treated (Ib), and vaccinated with dead vaccine (Id), respectively

IHC analysis of the cellular immune response of CD4^+^ and CD8^+^T cells exhibited a positive brown cell expression in all groups of the present work (Fig. 8). A slight immune expression of CD4^+^ and CD8 ^+^T cells was observed in the liver sections of the positive infected control mice (Ia). Hepatic sections of adjuvant-treated mice (Ib) displayed a mild expression of CD4^+^ and CD8^+^ T cells. However, significantly (*p* < 0.01) stronger and moderate cell infiltrations were recorded in the livers of the group vaccinated with killed tachyzoites (Id), and the group vaccinated with live attenuated tachyzoites (Ic), respectively, compared to the positive infected non-vaccinated control mice (Fig. 8).

## Discussion

The primary objective of our study is the development of a vaccine against toxoplasmosis. We studied the potential effect of gamma radiation attenuation of *Toxoplasma* tachyzoites isolated from the local virulent strain of *T.gondii* and a predominant genotype circulating in animals and human in Egypt, as a live attenuated vaccine on prevention of toxoplasmosis. Sheep are considered a major reservoir for *T*.*gondii* in many parts of the world and are often implicated in human infections due to the consumption of undercooked meat or contact with contaminated environments (Stelzer et al. 2019). While human isolates are important in understanding the disease in humans, our focus on sheep is driven by their epidemiological significance in zoonotic transmission. According to Buxton (1993)**,** the incomplete strain S48 (Toxoava) is the toxoplasmosis vaccine that is now on the market and is typically used in sheep to lower the abortion rate. However, it has some limitations that prevent its application on a large scale, also are not widely used or available in all regions (Zhang et al. 2015; Innes et al. 2019). There remains a need for improved vaccines, especially those that target different strains or provide broader immunity. The sheep isolate used in our study represents a virulent strain with characteristics similar to those that circulate in natural populations, which makes it an appropriate candidate for vaccine development in livestock. The RH HXGPRT (–) strain was selected for challenge experiments due to its well-established virulence and its utility in laboratory-based vaccine trials (Jensen et al. 2015). The HXGPRT gene knockout renders the strain resistant to certain selective agents, which facilitates tracking and analysis of the parasite's behavior in vivo. This genetically modified strain has been widely used in vaccine development studies, as it provides a consistent and reproducible model for evaluating immune responses and the effectiveness of potential vaccines.The use of this strain in our study ensures that we are working with a genetically defined and well-characterized strain, which is essential for accurately assessing the impact of our vaccine candidate.

The challenge dose of 2,500 tachyzoites (RH HXGPRT (–) strain) is relatively higher than natural exposure to infection but this dose does not necessarily reflect natural exposure levels, but is widely used in experimental *T.gondii* vaccine studies to test the upper limits of protection conferred by immunization **(**Ezz Eldin et al. 2015; Gaafar et al. 2022). The RH strain is known for its high virulence and rapid lethality in mice, making it is a suitable model for evaluating the protective strength of candidate vaccines. Using such a stringent challenge model allows researchers to detect even partial protective effects and provides a clear differentiation between effective and non-effective immunization strategies, which may not be apparent under milder, natural exposure conditions.

One challenge associated with the life cycle of *T.gondii* is its latent stage. Under immune system pressure, tachyzoites transform into slowly dividing bradyzoites within a tissue cyst. This transformation allows the parasites to evade the immune system and remain dormant until conditions are more favorable for reinfection (Rezaei et al. 2019). Notably, the RH virulent strain of *T.gondii* does not produce bradyzoites or form tissue cysts (Asgari et al. 2013). This characteristic is crucial for an ideal attenuated vaccine, as it ensures that no cysts remain in immunized individuals, thereby preventing the risk of parasite dissemination through vaccination. Consequently, we chose to use the virulent local strain, which likely addresses some of the limitations associated with the Pru and ME49 strains, such as the formation of cysts or suboptimal immunogenicity (Rezaei et al. 2019). The presented result has coincided with this discussion as the remaining live vaccinated mice after the challenge showed no cyst in the brain and other organs which was proved by examination of organs and the use of the mice homogenate to infect other mice however, no infection was developed.

Our previous research paper (El Shanawanyy et al. 2024a), concludes that the use of gamma irradiation application on a virulent strain of *T.gondii* at a dose of 0.25 kGy caused that parasite to still alive but it was attenuated and successfully elicited a specific immune response against toxoplasmosis, however did not infect mice. There were limited pathological changes observed in the liver and spleen, indicating a robust immune response following injection with these irradiated tachyzoites. These histopathological changes did not show necrosis in liver and spleen tissues, and tachyzoites were absent. In contrast, *T.gondii* infection typically results in obvious changes, such as clear cellular separation in the liver indicating hepatocellular dysfunction, and numerous necrotic cells in the spleen with absent lymphoid follicles (Zhuo et al. 2017). Furthermore, all mice remained alive until the end of the experiment. All of this information suggests that this live attenuated virulent strain is nearly harmless and safe for use as a vaccine candidate in mice. Furthermore, the use of 1.5 kGy did not cause infection but did lead to the death of tachyzoites with lower immune response. In this context, the present study was conducted to compare killed and live attenuated tachyzoite as a vaccine against acute toxoplasmosis.

In the present study, the body temperature of the mice was also measured to detect the side effects that may be caused by vaccines. The results reveal that there is no significant change in body temperature of the mice group injected with adjuvant (Ib) in comparison with control non-infected non-vaccinated mice after all doses of vaccines. However, group Ic showed a slight significant controlled elevation in body temperature after the first dose of vaccine, this result indicated that the vaccine is stimulating the immune system (Moscara et al. 2022; Finsterer et al. 2023). The significant and non-controlled increase in body temperature is a biomarker of toxic shock (Boles et al. 2003).

There are different researchers who use vaccine inoculation only once time (Yang et al. 2019; Xia et al. 2018; Xu et al. 2021), however, the use of three doses of inoculation results in enhanced response (Shen et al. 2023). So, we decided to inoculate the vaccine with three inoculation doses of both prepared vaccines live and killed. This was also, confirmed by our study (El Shanawany et al. 2024a) that proved that the immune response of live attenuated and killed tachyzoite would be decreased within one week.

The Survival rates determination in all mice (vaccinated and non-vaccinated) was used to determine the ability of protection against acute toxoplasmosis. In the present study, we compared the two vaccine candidates one is live attenuated irradiated tachyzoite at 0.25 kGy and the other is killed irradiated tachyzoite at 1.5 kGy the mice were vaccinated 3 times for two weeks with 2500 tachyzoite and then all mice challenged on day 45 with 2500 RH tachyzoites. Live attenuated vaccinated group showed statistically significant lowest mean number of tachyzoite count in the potential fluid when compared to the control infected group (Ia) (457.5 ± 61.51) with 99.8% reduction percentage and complete absence of tachyzoites in liver impression smear was recorded, this result was confirmed by histopathological examination of liver with complete absence of tachyzoites. Moreover, the mice-infected group all died within 7 days, while the use of live attenuated vaccine resulted in an increase in survival percentage to 57.1% with a mean survival time of 67.778 ± 26.4 the relatively high standard deviation observed in the live-attenuated vaccine group (Ic) reflects the extended survival of several animals compared to the majority. While the median survival time is slightly lower than the mean, this variation indicates that a subset of mice exhibited a remarkably prolonged response to the vaccine this reflect the increase in the SE value as median is 11 this mean that most of mice died at 11 day however the other mice still alive for long time of period results in increase the SE. This result supports the strong protective potential of the live-attenuated vaccine and highlights its ability to induce prolonged survival in challenged animals. Our results were supported by some way to Shen et al. (2023) who proved that RHΔ*ompdc*Δ*uprt* mutant live attenuated vaccine contributed 100% protection against the RHΔ*ku80*, ME49, and WH6 strains through a humoral and cellular mixed immune response in a murine model. Our vaccination protocol results proved better than Hiramoto et al. (2002) who showed that all control infected mice died after 7 days post-infection. However, all mice inoculated with exposed to 50, 100, or 200 Gy irradiated parasites at three doses for two weeks intervals, and then challenged all mice with10^3^ RH tachyzoites died after two weeks and, the presence of benign infection in some mice wasn't excluded, especially in groups which received tachyzoites irradiated at a lower dose. This information confirms our suggestion that the dose of irradiation should be selected carefully as the immunogenicity and protection levels are linked to the degree of attenuation of the parasites. Also, safety must be taken into account. The difference in result between our study and Hiramoto et al. (2002) may be related to changes in our vaccination protocol applying a longer post-vaccination period which affects the protection this is following Zorgi et al. (2016) who observed that the challenge with ME49 or VEG strain after a longer post-vaccination period of 90 days with RH irradiated tachyzoite at 0.255 kGy showed a significant decrease in the number of parasites in the brain tissue compared with the infected mice without vaccination.

IFN-γ and IL-12 were significantly increased after challenge in mice immunized with live attenuated vaccine in comparison with other groups of mice, which turn provided effective protection against toxoplasmosis induced by RH virulent strain. Correspondingly, the results showed that mice vaccinated with live attenuated vaccine exhibited protection at 99.8% against toxoplasmosis with increases in survival percentage to 57.1% with mean survival time (67.778 ± 26.4). Our result can be discussed as inoculation with live attenuated tachyzoites recruits neutrophils early and acts as an innate effector cell, resulting in the early secretion of IFN-γ and IL-12 to destroy intracellular parasites or inhibit their replication and proliferation (Gigley et al. 2009; Ivanova et al. 2016). The findings align with the study by Wang et al. (2020), where a CRISPR/Cas9-engineered *T.gondii* live attenuated strain (WH3 Δrop18) induced robust Th1 responses, marked by elevated IFN-γ and IL-12 production. This immune profile provided long-term immunity and protection against both virulent strains, supporting the hypothesis that Th1 cytokines are pivotal in mediating resistance to acute *T.gondii* infection. However, Mordue et al. (2001) and Nguyen et al. (2003) discussed the theory that excessive levels of Th1 inflammatory cytokines may result in excessive pathological damage and excessive levels of Th1 inflammatory cytokines may result in severe pathological damage and lead to death of the host. Therefore, regulatory cytokines are crucial for modulating the inflammatory response during *T.gondii* infection, promoting tissue regeneration **(**Bechara et al. 2021**)**. In addition, IL-17 can influence the behavior of other immune cells and non-immune cells, contributing to the regulation of immune responses. It can induce the production of other cytokines, chemokines, and inflammatory mediators, thereby amplifying and sustaining inflammatory reactions. Therefore, IL-17 is integral in maintaining the balance between effective immune defense and the prevention of excessive inflammation that can lead to tissue damage. Following this theory, our result revealed that IL 17 level was notably increased in response to live attenuated vaccine, and a high level was obtained after 6 days post-challenge which suggests control of infection and cause of increase in survival time. However, the level of IL 17 in mice vaccinated with killed tachyzoite showed a higher level than in control infected unvaccinated however, it is a low significant level in the group vaccinated with live attenuated vaccine which illustrates the low survival time in this group. Wang et al. (2020) similarly highlighted the role of CD4 + and CD8 + T cells in maintaining immunity, with IL-10 acting as a regulatory cytokine to prevent excessive inflammation. These findings underscore the delicate balance between pro-inflammatory and regulatory cytokines required for effective protection.

Forming pathogen-specific memory relies on T and B cells, which provide immune protection by recognizing antigen epitopes (Sasai et al. 2018; Khan and Moretto 2022). Humoral B cell immunity is vital for combating infection with toxoplasmosis. Antibodies can provide a protective immunity response by regulating parasite phagocytosis, invasion, and activating antibody-mediated classical complement pathway (Pifer and Yarovinsky 2011, Twfeek et al. 2023). In the present study a significant high level of anti-*T.gondii* IgG was noted in mice vaccinated with a live attenuated vaccine (Ic) after 7, and 16 days of vaccine, and remarkably increased after challenge at 6 days post-challenge in comparison with the control infected group and group vaccinated with killed prepared vaccine (Id). This result coincided with that of Wang et al. (2020), Shen et al. (2023), Li et al. (2020), Yang et al. (2019) who used other live attenuated *T.gondii* vaccines.

T-cell-mediated immune responses play a crucial role in preventing *T.gondii* invasion into host cells. Activation of CD4^ +^ T lymphocytes requires co-stimulators and MHC-II molecules, whereas CD8 ^+^  T cell activation depends on antigen-presenting cells or CD4 ^+^ T helper cells (Verdon et al. 2020). Our study revealed significant immune expression of CD4 ^+^ and CD8 ^+ ^T cells in liver sections of vaccinated mice. Specifically, there was a marked increase in CD8 ^+^ T cells in the live attenuated vaccine group (Ic) compared to the group vaccinated with killed tachyzoites (Id). These findings are consistent with previous studies, including Shen et al. (2023) and Seder et al. (2008), which emphasized the critical role of CD8 ^+^ T cells and IFN-γ in the control of *T.gondii* infection. Moreover, the study by Yang et al. (2019) also demonstrated the strong protective effect of an RH:1NPT1 mutant strain as live attenuated vaccine candidate, which elicited both cellular and humoral immunity. Supporting these findings, after vaccination using live attenuated *Toxoplasma* vaccine of WH3 Δ*rop18* Wang et al. (2020) showed that CD8^+  ^T cells play a critical role in resistance to intracellular pathogens, including *T.gondii*, through the secretion of IFN-γ, TNF-α, and cytotoxic particles. CD8^+^ T cells not only manage acute infection but also reduce intracerebral cyst burden in chronically infected mice. Similarly, CD4^+^ T cells are crucial in defense by secreting IFN-γ, a key cytokine in immunity against *T.gondii*. The novelty of our study lies in the successful application of a simple, low-cost attenuation method using gamma irradiation, as opposed to more complex and expensive technologies such as CRISPR/Cas9-based gene editing used by Wang et al. (2020). In addition, compared to Hiramoto et al. (2002), our modified vaccination protocol—with an extended interval between the final vaccine dose and the challenge, and a different irradiation dose led to enhanced antibody affinity and improved neutralization of *T.gondii* tachyzoites. These findings support the advancement of this approach as a practical and efficient strategy in the development of toxoplasmosis vaccines.

In conclusion, our study demonstrates that our prepared live attenuated tachyzoites offer a promising alternative for vaccine development against toxoplasmosis. The live attenuated vaccine triggered robust cellular and humoral immune responses, significantly enhancing survival time, and showed 57.1 % protection against acute toxoplasmosis, with substantial immune memory indicated by the sustained production of *Toxoplasma*-specific IgG antibodies, along with elevated levels of IFN-γ, IL-12, and IL-17 cytokines. Histopathological analysis further confirmed the absence of parasites in the liver and spleen of vaccinated mice, suggesting strong efficacy. Our results underscore the potential of gamma-irradiated tachyzoites as a safe and effective vaccine candidate, paving the way for the development of easy and time-consuming strategies in combating toxoplasmosis, a disease of significant global health concern. This study highlights a novel and cost-effective approach for developing a live attenuated toxoplasmosis vaccine using gamma irradiation, offering advantages over previously reported methods. Future directions may include exploring the vaccine’s efficacy in different animal models or under conditions of chronic infection could provide more translational insight.

## Data Availability

No datasets were generated or analysed during the current study.

## Declarations

### Reed health and safety

The laboratory health and safety procedures have been complied with within the course of conducting all experimental work reported in the current paper.
