# Correction: Comparative evaluation of live attenuated and killed tachyzoites as vaccine candidates for toxoplasmosis

**DOI:** 10.1186/s13568-025-01936-z

**Published:** 2025-09-11

**Authors:** Eman E. El Shanawany, Eman H. Abdel-Rahman, Waleed A. Nemr, Soad E. Hassan, Noha M. F. Hassan, Hassan M. Desouky, Rabab Zalat, Amany Ebrahim Nofal, Raafat M. Shaapan, Salwa Sami Younis

**Affiliations:** 1 Parasitology and Animal Diseases Department, Veterinary Research Institute, National Research Centre, Dokki, Giza, Egypt; 2 Department of Radiation Microbiology, National Center for Radiation Research and Technology, Egyptian Atomic Energy Authority, Cairo, Egypt; 3 Animal Reproduction and Artificial Insemination Department, National Research Centre, Dokki- Giza, Egypt; 4 Department of Parasitology, Theodor Bilharz Research Institute, Giza, Egypt; 5 Zoology Department, Faculty of Science, Histology and Histochemistry, Menoufia University, Shibin El-Kom, Egypt; 6 Department of Zoonosis, Veterinary Research Division, National Research Center, Dokki, Giza, Egypt; 7 Medical Parasitology Department, Faculty of Medicine, Alexandria University, Alexandria, Egypt

**Correction to: El Shanawany et al. AMB Express (2025) 15:102** 10.1186/s13568-025-01889-3

In the original publication of this article [1], the author noticed the errors in equation, Fig. 2 and Acknowledgments section. The corrections are listed below:

Incorrect equation:

Reductionrate (%) = Tachyzoite count of control infected group/ml − Tachyzoite count of control infected group/ml) /tachyzoite countofcontrol infected/ml) × 100.

Correct equation: 

Reduction rate (%) = [(Tachyzoite count of control infected group/ml−Tachyzoite count of treated group/ml)/Tachyzoite count of control infected group/ml]×100

In Funding section, the project number relating to National Research Centre given for Eman E. El Shanawany was incorrectly given as 1310125 and should have been 13010125.

In Fig. 2, part of the image overlaps with the caption of the figure. The correct image and caption are given below:

**Fig. 2:**
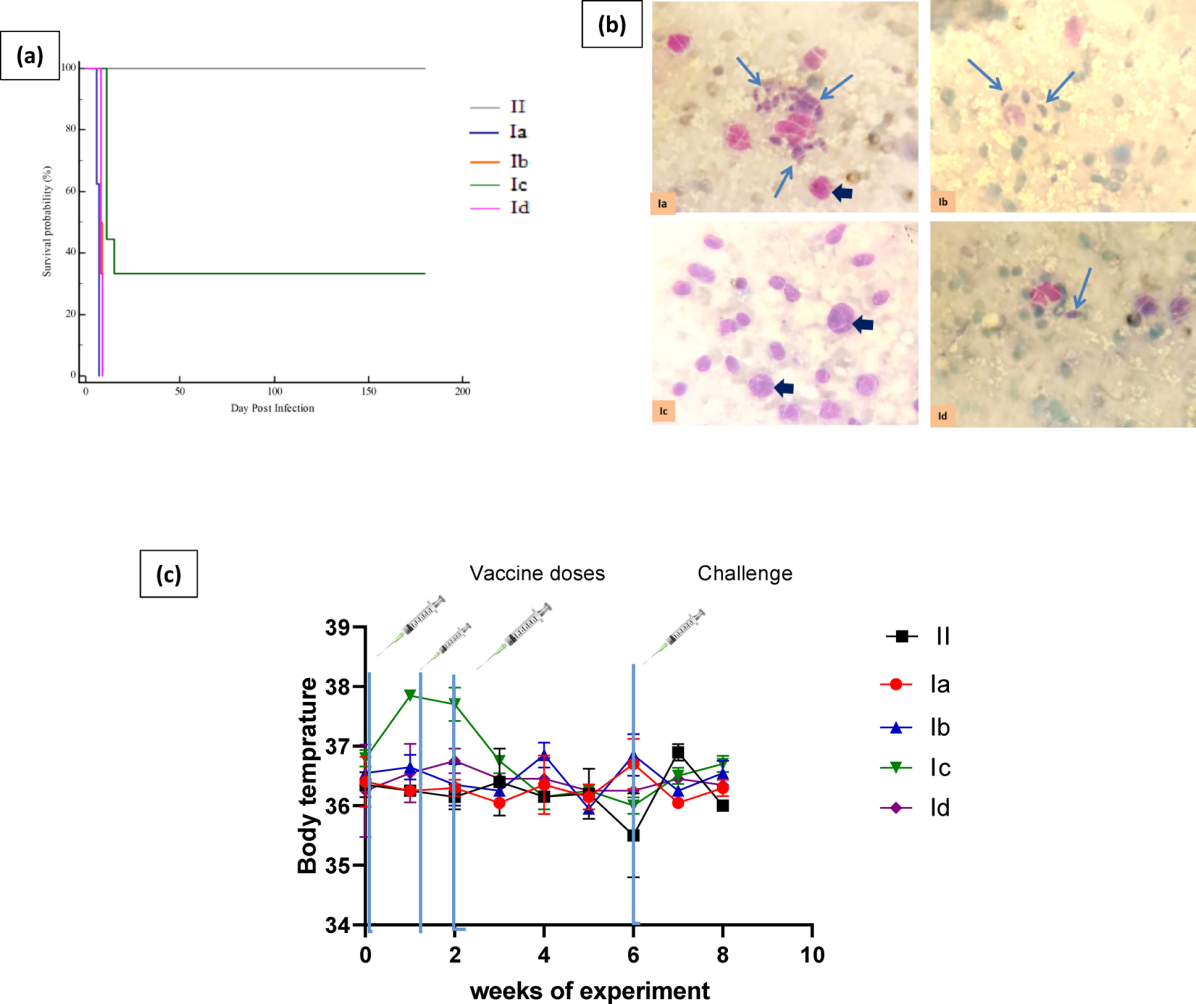
**a** Kaplan–Meier overall survival curve for the studied groups. **b** Liver impression smears from tested groups. **c** Rectal body temperature of the mice following during the experiment period. II: Control negative (non-infected non-vaccinated group). (Ia) control positive (Infected non-vaccinated group). Ib: Control group that received adjuvant complete and incomplete. Ic: Mice vaccinated with prepared live attenuated vaccine. Id: Mice vaccinated with the dead-prepared vaccine.  Free tachyzoites     Macrophages. Data are presented as means ± standard error
